# Image Reject Patterns in Computed Radiography: Insights From a Ghanaian Radiology Department

**DOI:** 10.1002/jmrs.70039

**Published:** 2025-12-04

**Authors:** Bismark Ofori‐Manteaw, Prosper Elinam Amevorwoshie

**Affiliations:** ^1^ Medical Radiation Science Discipline, School of Dentistry and Medical Science, Faculty of Science and Health Charles Sturt University Wagga Wagga New South Wales Australia; ^2^ Department of Radiology University of Ghana Medical Centre Accra Ghana

**Keywords:** computed radiography, image reject, quality assurance, reject analysis

## Abstract

**Introduction:**

Image reject analysis is a critical quality assurance (QA) tool in diagnostic imaging, helping to minimise unnecessary radiation exposure and improve imaging efficiency. This study evaluates image rejection patterns in a computed radiography (CR) system at a major tertiary teaching hospital in Ghana, identifying key sources of errors and their implications for radiology practice.

**Methods:**

A retrospective review of radiographic images acquired between April and June 2023 was conducted. Images, including those flagged as rejects were retrieved from the CR system and analysed for rejection rates, trends by anatomical region, and key error sources.

**Results:**

Of the 5889 images reviewed, 974 were rejected, resulting in an overall rejection rate of 16.5%. Rejection rates varied considerably across anatomical regions. High rejection rates were observed in skull/sinus (34.9%, *n* = 90/258), pelvic (29.9%, *n* = 88/294) and abdomen (26.9%, *n* = 84/312) examinations. Low rejects were recorded for ankle (1.8%, *n* = 2/110), humerus (2.4%, *n* = 2/82), forearm (6.7%, *n* = 6/90), elbow (9.7%, *n* = 6/62), and lower leg (7.5%, *n* = 16/214). Across all examinations, the three leading causes of image rejection were anatomical cut‐off (40.5%, *n* = 394), positioning errors (27.5%, *n* = 268), and beam centering errors (18.5%, *n* = 180). Less frequent causes included exposure‐related issues (6.6%, *n* = 64), patient movement (2.9%, *n* = 28), and artefacts or ghosting (4.1%, *n* = 40).

**Conclusion:**

This study reinforces the role of image reject analysis as a valuable QA measure in CR systems. The high rejection rates observed highlight the need for targeted interventions in positioning, workflow optimization, and radiographer training, particularly in resource‐constrained settings to enhance diagnostic quality and patient safety.

## Graphical abstract

This study examined image rejection patterns in a computed radiography system at a tertiary hospital in Ghana. An overall reject rate of 16.5% was observed, exceeding recommended thresholds, with anatomical cut‐off and positioning errors being the most common causes. The findings underscore the importance of targeted quality assurance strategies to improve imaging outcomes.

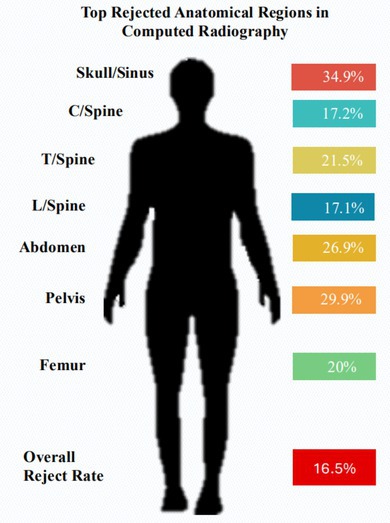

## 1 Introduction

Image reject analysis is a key quality assurance (QA) tool in diagnostic imaging. It helps radiology departments identify patterns in image rejection, reduce repeat exposures, and optimise workflow efficiency [1, 2, 3, 4, 5, 6]. High reject rates pose significant implications for healthcare facilities, particularly in resource‐constrained clinical settings where operational, infrastructural, and procedural challenges often persist [7, 8].

Despite the global shift towards digital radiography (DR), computed radiography (CR) remains widely used across many low‐ and middle‐income countries (LMICs), primarily due to its affordability and lower infrastructure demands [9]. Many hospitals in such settings only recently transitioned from analogue screen‐film systems and continue to rely heavily on CR for most general radiographic examinations [10, 11]. This delayed technological adoption highlights the need for sustained QA efforts tailored to CR, especially as image rejection remains a key concern. However, research on reject analysis and QA practices in CR within resource‐constrained environments is underexplored.

CR systems were introduced in Ghana more than two decades ago; however, anecdotal reports from clinical settings suggest that image rejection rates remain high, raising concerns about unnecessary radiation exposure and inefficiencies. There is also currently a lack of published data quantifying these reject rates in CR systems, highlighting the need for empirical investigation. Understanding the patterns and causes of image rejection in CR not only informs local practice but also provides insights relevant to other LMICs with similar radiographic infrastructures and challenges [11].

While training gaps and adaptation challenges in CR adoption have been widely reported [12, 13], several additional systemic factors contribute to high image rejection rates in resource‐constrained settings. These include aging equipment, inconsistent adherence to imaging protocols, insufficient QA oversight, and high patient throughput that places pressure on radiographers to perform under suboptimal conditions. In a study by Nyanthi et al. [14], 84% of radiographers reported lacking formal training in quality control procedures related to digital radiography, underscoring a significant educational gap.

In high‐volume clinical environments, time constraints, limited staffing, and workflow inefficiencies further hinder quality imaging practices and the ability to conduct regular reject audits [11]. Moreover, patient‐related factors such as motion artefacts, variations in body habitus, and cooperation difficulties can increase the risk of image rejection, particularly in complex or trauma‐related cases [15, 16]. The consequences of repeat imaging extend beyond operational inefficiencies; they contradict the ALARA (As Low As Reasonably Achievable) principle, increase radiation exposure, and disrupt departmental workflows [8, 11, 17, 18, 19, 20, 21]. Importantly, while errors such as anatomical cut‐off and poor positioning are often classified as “human‐related,” they are often symptoms of deeper systemic challenges [12, 13, 14]. Thus, even errors that appear individual in nature must be interpreted within the broader context of institutional constraints. Reject analysis in CR systems operating in these environments is therefore vital not only for identifying technical errors but also for informing targeted, systemic quality improvement strategies.

While reject analysis has historically been conducted in the context of screen‐film radiography (SFR), the transition to CR introduces new factors that may influence reject rates. The technological and workflow differences between CR and SFR such as exposure latitude, post‐processing capabilities, and digital review by radiographers can alter both the nature and frequency of image rejections [6, 8, 9, 22]. A study by Rosenkrantz et al. [23] reported that a considerable proportion of image rejections in digital systems may be inappropriate, suggesting that some repeats could be avoided with improved radiographer training. These distinctions make it essential to establish CR‐specific benchmarks for reject analysis, particularly in settings transitioning from analogue systems.

In high‐resource settings, reject analysis is often automated and integrated into digital systems that automatically collect and analyze data [16, 24, 25]. However, in many low‐resource contexts, including parts of sub‐Saharan Africa, reject audits are still conducted manually and inconsistently [24]. While literature provides data on reject rates and causes in SFR systems, relatively little is reported on CR systems in these regions. In Ghana, while some earlier studies focused on SFR, these do not reflect current CR‐based workflows or quality challenges [21, 26]. This study aimed to establish baseline data on CR reject rates in a major tertiary hospital in Ghana, addressing gaps in training, equipment maintenance, and protocol adherence.

## 2 Method

### 2.1 Study Design and Setting

This study was a quantitative retrospective analysis conducted at a tertiary health facility's imaging unit. It focused on X‐ray images acquired between April and June 2023 to determine the overall image reject rate, reject rates by anatomical regions and reasons for rejection. The 3‐month period was chosen as it reflects one of the busiest operational windows for the imaging department, marked by high patient throughput and a wide range of examinations, ensuring a representative sample of routine clinical imaging workload. While the number of images analysed may be deemed moderate compared to large‐scale international studies, it reflects the actual imaging volume of a radiology unit operating in a resource‐constrained environment. Additionally, this timeframe provided a feasible window for retrospective data retrieval without disrupting ongoing clinical workflow. Indeed, several international studies [1, 3, 8, 9, 20, 23, 27] have all reported a study period covering 2–4 months with some employing moderate sample sizes of about 4158 images. Moreover, our study prioritised depth of review and accuracy in manually verified image rejection data over sheer volume. The findings provide valuable baseline data in a context, where empirical data on CR rejection is lacking. Ethical approval for the study was obtained from the University of Health and Allied Sciences Research Ethics Committee (UHAS‐REC A.8 [142] 22–23).

The radiographic examinations in this study were conducted using a floor‐mounted Shimadzu CR X‐ray machine. This floor‐mounted unit is equipped with a fixed erect bucky and tabletop bucky, allowing for a broad range of imaging projections. The X‐ray machine uses Fuji CR imaging plates of varying sizes for various examinations, which are processed using a Fuji Capsula XLII image reader. The image acquisition and display are managed using Fuji's APL Software B V8.3.0001 (Image Intelligence), which is installed on a display monitor linked to the image reader. This software contains post‐processing tools that allow radiographers to view, manipulate, annotate, and analyse processed images. All X‐ray images from the set period were included in the analysis.

In this study, the expected average reject rate was guided by benchmarks commonly cited in the literature [5, 16, 24, 25]. The American Association of Physicists in Medicine (AAPM) Report No. 151 cites a typical range of 4% to 8% for reject rates in digital imaging departments including CR [1, 9, 24, 25, 28, 29, 30], and recommends 8% as a target reject rate and 10% as a threshold for initiating investigation and corrective action [3, 16, 31]. While these values are not absolute and may vary across clinical settings, they serve as useful benchmarks in evaluating CR performance and guiding local QA efforts [3, 16].

### 2.2 Data Collection and Extraction

The CR system used in the hospital facility allows radiographers to label rejected images and assign predefined reasons for rejection when an image is considered diagnostically unacceptable. These standardised rejection options are embedded in the CR system, ensuring uniform classification across all examinations within the facility. In the CR system used at the facility, rejected images are not deleted. Instead, they are stored within the system archive, along with the assigned rejection reason. This allows for retrospective audit and quality control review. These images were accessed during data extraction and were not manipulated or reassigned by the researchers.

Data were retrospectively collected after routine working hours over a two‐week period by an experienced radiography educator. To reduce the risk of extraction errors, data were systematically collected using a structured template. The template recorded the type of examination, number of images acquired, number of rejected images, and the assigned rejection reasons. No new labels or categories were introduced by the researchers, and they were not involved in assigning any of the rejection reasons. All extracted data were reviewed and verified against the source system and double‐checked for completeness and accuracy. Any unclear entries or inconsistencies were resolved by the research team before analysis.

### 2.3 Data Analysis

Data were analysed using Microsoft Excel (version 2021). Descriptive statistics, including frequencies and percentages, were used to summarise the total number of images acquired, number of rejected images, rejection rates per examination type, and reasons for rejection. The rejection rate for each anatomical region was computed by dividing the number of rejected images of the anatomical region by the total number of images acquired for that region (Table 1). The causes of image rejection were categorised as follows: A = exposure error, B = positioning error, C = patient movement, D = anatomical cut‐off, E = beam centering error, and F = artefact/image ghosting (Table 2). The proportion of each rejection cause was determined by dividing the frequency of that specific reason by the total number of rejected images recorded.

**TABLE 1:** Image reject rates.

Examinations	Number of images (*α*)	Number of rejects (*β*)	Reject rate (*β*/*α*)	Level of complexity
Lumbar spine	1134	194	17.1%	HC
Chest	1422	180	12.7%	MC
Skull/sinuses	258	90	34.9%	HC
Pelvis	294	88	29.9%	HC
Abdomen	312	84	26.9%	HC
Thoracic spine	158	34	21.5%	HC
Cervical spine	348	60	17.2%	HC
Shoulder	164	22	13.4%	MC
Humerus	82	2	2.4%	LC
Elbow	62	6	9.7%	LC
Forearm	90	6	6.7%	LC
Wrist	126	14	11.1%	LC
Hand	78	12	15.4%	LC
Femur	295	58	20.0%	HC
Knee	586	82	14.0%	MC
Lower leg	214	16	7.5%	LC
Ankle	110	2	1.8%	LC
Foot	156	24	15.4%	MC
Total	5889	974	16.5%	

Abbreviations: HC, high complexity; LC, low complexity; MC, medium complexity.

**TABLE 2:** X‐ray examinations and corresponding causes of rejects.

Examinations	Reasons for reject					
	A	B	C	D	E	F
Lumbar spine	32	46	4	48	46	18
Chest	4	22	22	108	24	0
Skull/sinuses	6	40	0	36	4	4
Pelvis	2	18	0	52	12	4
Abdomen	4	20	0	42	18	0
Thoracic spine	0	8	2	14	10	0
Cervical spine	2	32	0	14	8	4
Shoulder	0	8	0	6	6	2
Humerus	0	0	0	2	0	0
Elbow	0	6	0	0	0	0
Forearm	0	2	0	0	4	0
Wrist	0	6	0	4	4	0
Hand	0	0	0	12	0	0
Femur	4	8	0	26	14	6
Knee	10	36	0	20	14	2
Lower leg	0	10	0	4	2	0
Ankle	0	2	0	0	0	0
Foot	0	4	0	6	14	0
Total	64	268	28	394	180	40

*Note:* A: exposure; B: positioning error; C: patient movement; D: anatomical cut‐off; E: beam centering error; F: artefact/image ghosting.

To better contextualise the variation in image rejection rates, examinations were grouped into three categories based on their procedural and anatomical complexity: high, medium and low complexity. Classifications were informed by literature and professional clinical practice guidelines [9, 24, 32, 33]. Chi‐square tests, Kendall's tau‐b, and Spearman's rho correlations were performed to examine the association and strength of relationships between examination complexity levels, image rejection rates and the frequencies of specific rejection causes.

## 3 Results

A total of 5889 radiographic images were acquired for 2795 patients. 1332 (47.7%) of the patients were males and 1463 (52.3%) were females. Chest and lumbar spine were the most frequently performed examinations, representing 24.1% and 19.3% of the total images reviewed.

### 3.1 Overview of Reject Rates

Of the 5889 images reviewed, 974 were flagged rejected, resulting in an overall rejection rate of 16.5%. The median number of images acquired per examination type was approximately 294 (range: 62–1422). Rejection rates varied considerably across anatomical regions. High rejection rates were observed in skull/sinus (34.9%, *n* = 90/258), pelvic (29.9%, *n* = 88/294) and abdomen (26.9%, *n* = 84/312) examinations. Other regions with notable rejects include thoracic spine (21.5%, *n* = 34/158), lumbar spine (17.1%, *n* = 194/1134), femur (20.0%, *n* = 58/295) and chest (12.7%, *n* = 180/1422). Among all anatomical regions examined, the lowest rejection rates were recorded for ankle (1.8%, *n* = 2/110), humerus (2.4%, *n* = 2/82), forearm (6.7%, *n* = 6/90), elbow (9.7%, *n* = 6/62), and lower leg (7.5%, *n* = 16/214).

### 3.2 Distribution of Rejection Causes

**FIGURE 1:**
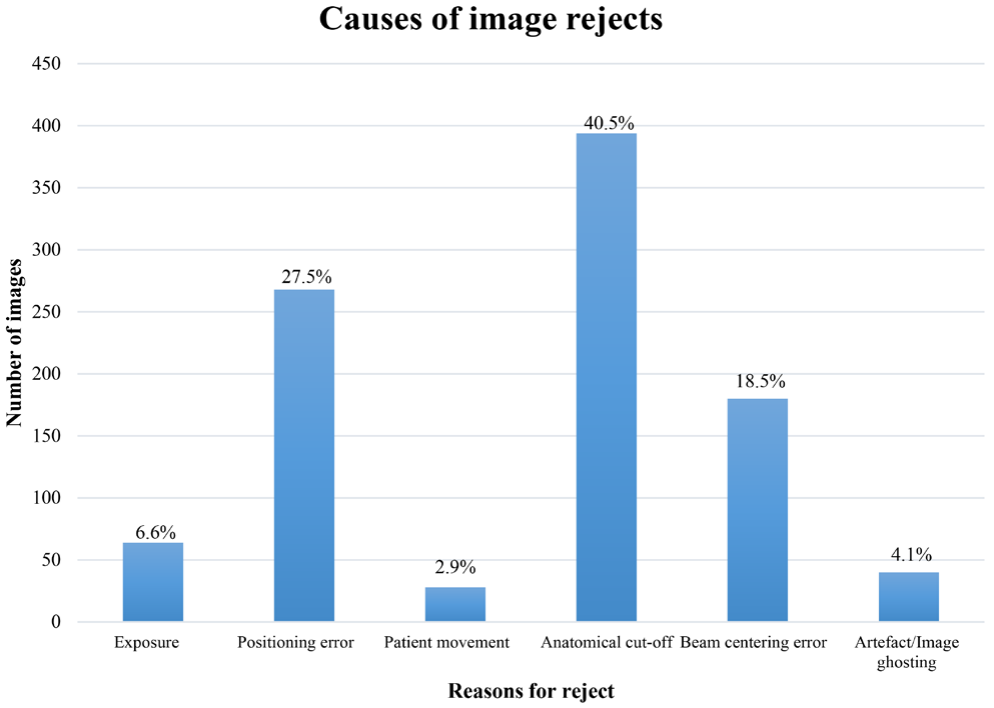
Distribution of image rejection causes.

Across all examinations, the three leading causes of image rejection were anatomical cut‐off (40.5%, *n* = 394), positioning errors (27.5%, *n* = 268), and beam centering errors (18.5%, *n* = 180). Less frequent causes included exposure‐related issues (6.6%, *n* = 64), patient movement (2.9%, *n* = 28), and artefacts or ghosting (4.1%, *n* = 40). These are summarised in Figure 1 and Table 2.

### 3.3 Reject Trends by Complexity of Examination

The complexity levels of examinations were used to explore associations in reject rates. Examinations such as lumbar, cervical, thoracic, skull and pelvic were classified as high complexity due to their challenging positioning requirements and the critical need for anatomical precision. Examinations such as ankle, forearm, and elbow were classified as low complexity owing to their relatively straightforward positioning and reduced dependency on patient cooperation (Table 1). The Chi‐square test showed a statistically significant association between examination complexity and frequency of specific rejection causes [χ^2^(10) = 62.03, *p* < 0.001]. Both Kendall's tau‐b and Spearman's rho non‐parametric tests demonstrated strong, statistically significant positive correlations between examination complexity and reject rate. Specifically, Kendall's tau‐b yielded a coefficient of 0.776 (*p* < 0.001), while Spearman's rho indicated an even stronger association with a coefficient of 0.893 (*p* < 0.001). Table 3 provides detailed information on examination complexity and frequency of reject causes.

**TABLE 3:** Examination complexity and reject cause cross tabulation.

Reject cause								
		Anatomical cut‐off	Beam centering	Exposure	Image ghosting	Patient movement	Positioning	Total
HC	Count	232	112	50	36	6	172	608
	% within examination	38.2%	18.4%	8.2%	5.9%	1.0%	28.3%	100.0%
	% within reject cause	58.9%	62.2%	78.1%	90.0%	21.4%	64.2%	62.4%
LC	Count	22	10	0	0	0	26	58
	% within examination	37.9%	17.2%	0.0%	0.0%	0.0%	44.8%	100.0%
	% within reject cause	5.6%	5.6%	0.0%	0.0%	0.0%	9.7%	6.0%
MC	Count	140	58	14	4	22	70	308
	% within examination	45.5%	18.8%	4.5%	1.3%	7.1%	22.7%	100.0%
	% within reject cause	35.5%	32.2%	21.9%	10.0%	78.6%	26.1%	31.6%
Total	Count	394	180	64	40	28	268	974
	% within examination	40.5%	18.5%	6.6%	4.1%	2.9%	27.5%	100.0%
	% within reject cause	100.0%	100.0%	100.0%	100.0%	100.0%	100.0%	100.0%

Abbreviations: HC, high complexity; LC, low complexity; MC, medium complexity.

## 4 Discussion

### 4.1 Overview of Reject Rates

Although CR systems are expected to yield lower image reject rates due to their broad dynamic range and post‐processing flexibility [8, 9], the overall reject rate of 16.5% observed in this study surpasses the recommended threshold of 8%–10% and those reported in similar studies [1, 6, 16, 19, 20, 34, 35, 36]. This finding is surprisingly consistent with earlier studies in Ghana using screen‐film radiography which reported rates of 14.1% and 19.9% [21, 26]. The persistence of high reject rates despite a shift to CR suggests that digitisation alone does not resolve underlying issues, particularly in resource‐constrained settings. While our study did not directly evaluate contributing factors such as training adequacy or equipment issues, the results align with previous research that has associated similar reject patterns with such systemic challenges [1, 12, 14, 19].

### 4.2 Anatomical Variability in Reject Rates

The findings reveal substantial variability in reject rates across different anatomical regions, with complex anatomical regions showing high rejects. In this study, reject rates were strongly correlated with examination complexity levels. These findings suggest that as the complexity of the radiographic examination increases, so does the likelihood of image rejection. While skull/sinus and pelvic examinations recorded the highest reject rates, other examinations such as the abdomen, cervical, thoracic and lumbar spines also exceeded the overall average of 16.5%, highlighting broader quality assurance challenges. The disproportionately high rejection rates observed in complex examinations are consistent with the technical demands and positioning challenges associated with these regions [9, 24, 33]. Skull/sinus exams are prone to errors in beam angulation, head tilt, and external marker placement. The complexity of visualising intricate bony structures and the need for multiple projections increases the likelihood of image repeats [9, 33]. Furthermore, the need for precise grid use and high‐detail resolution, along with patient movement, contributes to the challenge of producing diagnostic‐quality images on the first attempt [9, 10].

Pelvic and femur imaging often suffers from improper leg rotation and beam misalignment, which are further exacerbated in trauma cases [9, 17]. In trauma cases, where immobilisation devices or acute pain limit movement, achieving the correct anatomical position can be even more challenging, further increasing repeat rates [17]. To address these challenges, targeted interventions such as enhanced radiographer training on complex anatomical positioning, the use of positioning aids, stricter adherence to imaging protocols, and quality control audits should be prioritised.

Abdominal exams showed high repeat rates due to position, anatomical and beam centering errors. Also, motion artefacts, variable soft tissue densities, and poor breath‐hold compliance by patients during imaging further compromise contrast resolution and anatomical delineation [22]. Cervical, thoracic and lumbar spine radiography also recorded high reject rates. Although their frequency of use may be decreasing in some countries due to the wide availability of Computed Tomography or Magnetic Resonance Imaging, they are common examinations in our local setting. The lateral views of the spine are especially challenging, requiring precise centering and alignment despite spinal curvature or body habitus [17, 20, 37].

Although chest radiography is widely considered technically simple [17], it recorded a relatively high rejection rate in our context. This may be attributed to the challenges encountered when imaging non‐ambulatory or uncooperative patients [9, 17]. Factors such as poor scapular positioning, suboptimal inspiration, and beam angulation errors frequently result in repeated exposures [9]. These clinical complexities, rather than technical limitations of the equipment, could account for the high rejection rate in this context. Thus, it is imperative that reject analysis extends beyond traditionally complex anatomical regions to encompass other high‐reject areas like the chest, abdomen and spine.

The relatively low rejection rates in ankle, humerus, forearm, elbow, and lower leg imaging may be attributable to simpler positioning techniques, clearly defined anatomical landmarks, and reduced dependence on patient cooperation, all of which minimise the potential for errors [24]. These areas typically involve straightforward beam alignment, predictable collimation, and standard exposure factor selection [9]. Additionally, patients undergoing these examinations are often ambulatory and more compliant, reducing the likelihood of motion artefacts. These low rejection rates align with findings from the large‐scale study conducted by Foos et al. [24].

In this study, high‐complexity examinations accounted for most rejected images, reinforcing the notion that anatomical and procedural difficulties associated with these examinations significantly influence rejects. These findings provide evidence for prioritising targeted QA efforts and radiographer training in high‐complexity examinations.

### 4.3 Causes of Image Rejection

**FIGURE 2:**
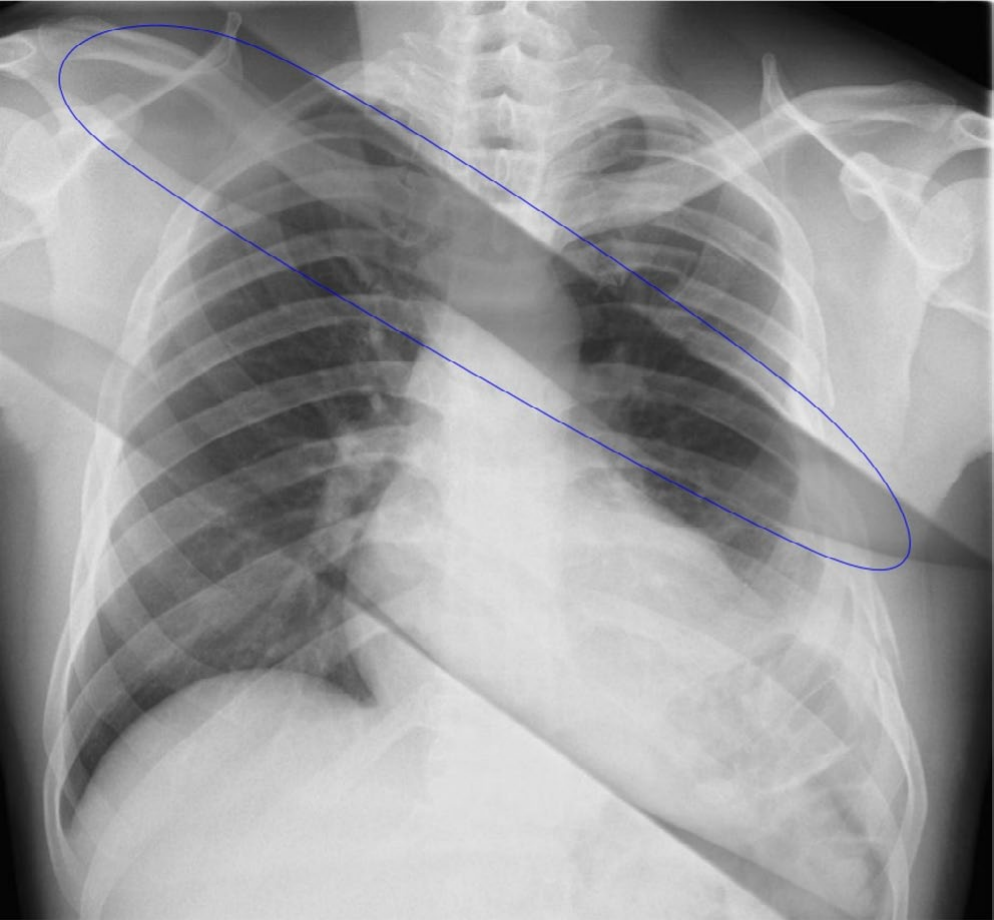
A chest radiograph demonstrating diagonal artefacts across the thoracic region (circled blue). These artefacts, likely due to ghosting or image plate memory effect, appear as faint, dark bands traversing the lung fields. Such artefacts are typically associated with incomplete erasure of the CR imaging plate, resulting in residual signals from previous exposures being superimposed on new images [38].

Across all examinations, three error types predominated: anatomical cut‐off, positioning errors and beam centering errors. Together, these accounted for 86.5% of all rejects (Figure 1) mirroring findings from prior studies in digital systems [8, 24, 27, 28, 29]. Other causes included exposure‐related factors, patient movement and image artefact/ghosting (Figure 2).

**FIGURE 3:**
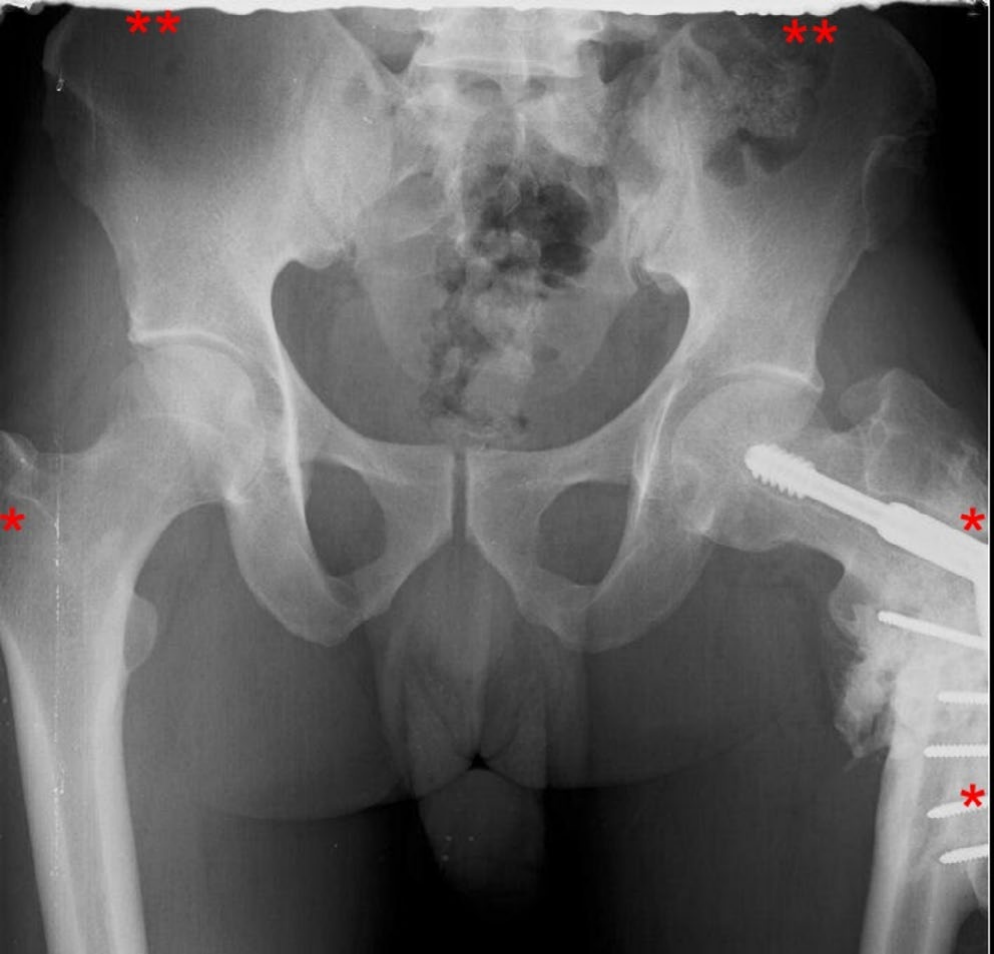
Pelvic radiograph demonstrating anatomical cut‐off. Critical anatomical landmarks including the superior margins of the iliac crests, both greater trochanters, and the proximal third of the left femur are not fully visualised within the field of view (*regions annotated in red* *). This omission renders the image non‐diagnostic for comprehensive pelvic and hip evaluation.

Anatomical cut‐off (Figure 3), positioning and beam centering errors point to difficulties in achieving accurate patient alignment and field of view coverage, particularly for examinations with complex projections or variable anatomy. In a large‐scale CR‐focused study by Foos et al., positioning errors and anatomical cut‐off together contributed to 45% and 56% of rejections at a community hospital and a university hospital respectively [8, 24]. These issues are amplified in high‐throughput or emergency settings, where time constraints, communication barriers and patient cooperation may be limited [8]. Motion blur, breath‐hold difficulties, and pain‐related restricted mobility further compound the risk of image repeats, especially for abdominal, thoracic, and spine examinations particularly in emergency and inpatient settings [16]. Although exposure‐related rejects were relatively low, likely due to the CR system's wider exposure latitude and post‐processing abilities [4, 9, 28, 29, 30], their presence indicates that exposure optimisation protocols still need reinforcement.

A chi‐square analysis revealed a statistically significant association between examination complexity levels and the frequencies of image rejection causes [χ^2^(10) = 62.03, *p* < 0.001]. This finding suggests that the types of errors leading to image rejection are influenced by the complexity of the procedure. Table 2 offers deeper insights into how anatomical complexity influences error patterns. High‐complexity examinations accounted for a disproportionately large number of positioning errors and anatomical cut‐off issues, further underscoring the difficulties in accurately localising and imaging complex anatomical regions. Moderate‐complexity examinations displayed mixed rejection patterns. For instance, the knee recorded 36 positioning and 20 anatomical cut‐off errors, while the femur presented 8 and 26 respectively. These findings suggest that even moderately complex examinations are vulnerable to repeat imaging when optimal positioning is not achieved. In contrast, lower‐complexity extremity examinations showed minimal rejection counts across all error types. This pattern reinforces the strong association between anatomical and procedural complexity and the frequency of image rejections, emphasising the need for targeted quality assurance measures in high‐complexity imaging procedures.

### 4.4 Quality Assurance Strategies and Implications for Practice

The continued use of CR systems across many LMICs, particularly in institutions that have only recently transitioned from analogue SFR underscores the importance of this study's findings. While digital systems such as CR offer significant advantages over SFR, our results demonstrate that digitalisation alone does not inherently resolve image rejection challenges. In fact, the persistently high rejection rates observed in this study point to deeper, multifactorial issues involving radiographer performance, workflow constraints, and system‐level inefficiencies. This highlights the need for targeted, context‐sensitive QA strategies that extend beyond equipment upgrades. Facilities operating in resource‐constrained environments must prioritise quality improvement initiatives that are both cost‐effective and operationally feasible within the realities of their settings. In these settings, where CR systems remain widely used, reject audit offers a valuable, cost‐effective approach for monitoring performance, identifying error patterns, and informing both clinical governance and professional development programmes.

To address the challenges observed, radiographer‐focused strategies should include structured training on advanced positioning techniques as well as simulation‐based practice for trauma and non‐cooperative patient scenarios [10]. These educational strategies should be embedded into both pre‐service education and continuing professional development (CPD) programmes [9].

At the workflow and system level, implementing standardised imaging protocols, pre‐exposure checklists, and real‐time positioning verification could significantly reduce preventable errors. Dedicated imaging time slots for trauma or emergency cases may also allow radiographers to manage difficult cases without compromising image quality due to time constraints. Moreover, consistent equipment calibration, preventative maintenance, and timely software updates are essential to reduce rejects, especially where specialised dedicated imaging units (e.g., paediatric or A&E) are unavailable.

A critical aspect of any QA initiative is ongoing performance monitoring. Regular reject audits targeting high‐frequency error categories can offer timely feedback loops and promote reflective practice among radiographers [9, 10]. Integration of audit outcomes into CPD frameworks can reinforce accountability while fostering a culture of continuous quality improvement. Ultimately, while high‐income settings have largely transitioned to DR systems, the operational reality in many LMICs necessitates optimised QA strategies tailored specifically for CR. The findings of this study reaffirm that improving radiographic quality and minimising unnecessary radiation exposure requires a multifaceted approach, one that aligns radiographer education, workflow design, and institutional governance with the practical constraints of resource‐limited healthcare systems.

## 5 Limitations

This study has several limitations that should be acknowledged. First, its retrospective design meant that the researchers were not directly involved in assigning the reasons for image rejection at the time of acquisition. Instead, rejection reasons were selected by radiographers from predefined system‐generated options, introducing the potential for subjectivity and inter‐operator variability [22]. This may have led to inconsistencies in how rejection causes were categorised. Second, the study was conducted at a single facility. As such, the findings may not be generalizable to other institutions with differing workflows, equipment, or staffing levels. Broader multi‐centre studies would be needed to validate and extend these results across more diverse clinical environments. Third, the analysis did not take into consideration patient or radiographer‐related factors. Despite these limitations, the study provides valuable baseline insights into CR image reject patterns within a high‐volume, resource‐constrained setting. The findings may inform targeted QA initiatives and support broader discussions on radiography practice improvement in similar LMIC contexts.

## 6 Conclusion

This study underscores the importance of routine reject analysis in CR settings as a key QA strategy, particularly within resource‐constrained settings. This study highlights a high image rejection rate in a CR system, exceeding recommended thresholds, highlighting persistent technical and systemic challenges despite the transition from SFR. Positioning‐related errors, anatomical cut‐offs and beam centering errors emerged as the leading causes of image rejects. Reducing reject rates in CR requires a multifaceted approach, including radiographer education, protocol standardisation, sustained QA programs, and system‐level interventions. By addressing these factors, imaging departments can enhance image quality, minimise unnecessary radiation exposure, and improve diagnostic efficiency.

## Conflicts of Interest

The authors declare no conflicts of interest.

## Data Availability

The data that support the findings of this study are available from the corresponding author upon request.
